# The Core Concepts, Competencies, and Grand Challenges of Comparative Vertebrate Anatomy and Morphology

**DOI:** 10.1093/iob/obac019

**Published:** 2022-07-30

**Authors:** Nicole Danos, Katie Lynn Staab, Lisa B Whitenack

**Affiliations:** Biology, University of San Diego, 5998 Alcala Park, San Diego, CA 92210, USA; Biology Department, McDaniel College, 2 College Hill, Westminster, MD 21157, USA; Depts. of Biology and Geology, Allegheny College, 520 N. Main St., Meadville, PA 16335, USA

## Abstract

Core concepts offer coherence to the discourse of a scientific discipline and facilitate teaching by identifying large unifying themes that can be tailored to the level of the class and expertise of the instructor. This approach to teaching has been shown to encourage deeper learning that can be integrated across subdisciplines of biology and has been adopted by several other biology subdisciplines. However, Comparative Vertebrate Anatomy, although one of the oldest biological areas of study, has not had its core concepts identified. Here, we present five core concepts and seven competencies (skills) for Comparative Vertebrate Anatomy that came out of an iterative process of engagement with the broader community of vertebrate morphologists over a 3-year period. The core concepts are (A) evolution, (B) structure and function, (C) morphological development, (D) integration, and (E) human anatomy is the result of vertebrate evolution. The core competencies students should gain from the study of comparative vertebrate anatomy are (F) tree thinking, (G) observation, (H) dissection of specimens, (I) depiction of anatomy, (J) appreciation of the importance of natural history collections, (K) science communication, and (L) data integration. We offer a succinct description of each core concept and competency, examples of learning outcomes that could be used to assess teaching effectiveness, and examples of relevant resources for both instructors and students. Additionally, we pose a grand challenge to the community, arguing that the field of Comparative Vertebrate Anatomy needs to acknowledge racism, androcentrism, homophobia, genocide, slavery, and other influences in its history and address their lingering effects in order to move forward as a thriving discipline that is inclusive of all students and scientists and continues to generate unbiased knowledge for the betterment of humanity. Despite the rigorous process used to compile these core concepts and competencies, we anticipate that they will serve as a framework for an ongoing conversation that ensures Comparative Vertebrate Anatomy remains a relevant field in discovery, innovation, and training of future generations of scientists.

## Introduction

The study of anatomical structures is one of the oldest subdisciplines in the biological sciences (Cosans and Frampton 2015; Wanniger 2015) and has contributed to our understanding of organismal function and given us insight into how phenotypes develop, function, and evolve. Vertebrate Morphology today is a foundational and thriving biological science. For example, out of the Society for Integrative and Comparative Biology's (SICB) 5289 members, roughly 10% are members of the Division of Vertebrate Morphology (DVM) (M. Johnson 2022, pers. comm.). Similarly, the American Association for Anatomy is a thriving organization that publishes work in basic anatomy (The Anatomical Record), development (Developmental Dynamics), and education (Anatomical Sciences Education) and supports a diverse body of scientists.

We are still discovering new anatomical structures (e.g., Benias et al. 2018; Siomava et al. 2020) and technological advances in imaging allow for unprecedented visualization and quantification of vertebrate anatomy, its variation, physiological properties, and morphology in both anatomical specimens and in action (e.g., XROMM [e.g., Brainerd et al. 2010; Knörlein et al. 2016], diceCT [e.g., Gignac et al. 2016], musculoskeletal computer models [e.g., Buser et al. 2020; Demuth et al. 2020], synchrotron X-ray imaging [e.g., Vallcorba et al. 2021], geometric morphometrics [e.g., Klingenberg 2011; Zelditch et al 2012]). Together with analytical tools such as finite-element analysis (e.g., Dumont et al. 2005; Polly et al. 2016), fluid dynamics modeling (e.g., Tytell et al. 2010), and gait analysis (e.g., Reilly and McElroy 2007), these quantitative morphological data have led to breakthroughs in our understanding of the basic relationship between form and function in vertebrates, leading in turn to a deeper understanding of how vertebrate anatomy and morphology have evolved (for a comprehensive example see Chapter 3 “Major transformations in vertebrate breathing mechanisms” by E.L. Brainerd in “Great Transformations in Vertebrate Evolution”) and how these principles can inspire engineering solutions.

Practically speaking, for students who go on to enter health careers, Anatomy is the basis of clinical examinations, surgeries, physical therapy, and diagnostic imaging technologies. For students who go on to biomedical research, a working knowledge of Comparative Anatomy will be critical as they interpret data from model organisms for application to human health. But all students will be healthcare consumers and stewards of our natural world; having a working knowledge of the fundamentals at the core of the Anatomical and Morphological Sciences will be critical for making informed choices with potentially wide-ranging impacts as voters and consumers.

The comparative aspect of anatomical study was critical from early on in establishing structure–function relationships. Aristotle used the comparative approach to identify the characteristics of all mammals (Aristotle 350 BCE). The resurgence of comparative anatomy in the late 18th and 19th centuries by the likes of Georges Cuvier, Mary Anning, and Richard Owen, equipped Darwin and Wallace with the support they needed to outline their evolutionary theories: the form of animals is dictated by the struggle for existence (form-function) but also by their shared ancestry (descent with modification) (Cosans and Frampton 2015; Blits 1999). Intra- and interspecific variation are central to the identification of these ideas. In fact, modern comparative methods provide a mathematical framework to test for the strength of form–function relationships while taking shared ancestry into consideration (review in Felsenstein 1985; Huey et al. 2019).

Throughout this manuscript, we define the anatomy of an organism as the sum of its body parts and structures. We define the morphology of an anatomical structure as the sum of those characteristics (shape, size, texture, etc.) that describe its form (Greek μορφή, morphe) of that structure (Wenzel and Zaharia 2012). These same words often also refer to the study of comparative vertebrate anatomy and morphology (Miriam Webster dictionary; AAA website). Therefore, we capitalize Anatomy and Morphology when we refer to the scientific fields, and use lower case anatomy and morphology when they refer to a specific organism or structure. For the rest of this manuscript, we use the term Comparative Vertebrate Anatomy to include the field of Vertebrate Morphology.

As the fields of Comparative Vertebrate Anatomy have grown by leaps and bounds in recent years, so has the Scholarship of Teaching and Learning (SoTL). As teachers, we are all more aware of the impact our pedagogical strategies in the classroom, in the laboratory, and in the field can have on student success and retention. For example, a number of studies show that active learning and inquiry-based pedagogies are very effective in biology classrooms (e.g., Armbruster et al. 2009; Haak et al. 2011; Freeman et al. 2014). SoTL studies that address human anatomy call for understanding over memorization (e.g., Miller et al. 2002; Morris 2015) and for “authentic learning” where content is explicitly linked to students’ lives (e.g., clinical practice in Pawlina and Drake 2016; case studies in Anstey 2017). These pedagogical approaches can take more classroom time to implement than traditional lectures at the expense of content (e.g., Walker et al. 2008). Moreover, recommendations for science education reform in undergraduate biology education, such as reports from the NRC (2003) and AAAS (2011), call for a focus on student understanding and use of disciplinary core principles, not just memorization of information. Although there are entire journals dedicated to the teaching of medical/human anatomy to help those instructors navigate these recommendations, the pedagogical literature on teaching Comparative Vertebrate Anatomy is generally lacking. Since the 2011 AAAS publication “Vision and Change”, core concepts have been defined for molecular biology and biochemistry (Tansey et al. 2013), plant biology (ASPB-BSA), physiology (Michael et al. 2017), ecology (Ecological Society of America 2018), and microbiology (Merkel et al. 2012). However, the core concepts and competencies of Comparative Vertebrate Morphology have not been established.

Anatomy and Morphology have also reflected the beliefs and values of the practicing scientists in their time. The contributions from and dependence on a diverse community that have supported the field from the beginning have not been well acknowledged, nor has the connection of Comparative Anatomy and Morphology to racism and eugenics. Addressing diversity and incorporating anti-racist pedagogy is necessary in order to eliminate blind spots in our field's growth and to reduce barriers to entry and professional success for subsections of the population that have been historically excluded.

In this manuscript, we aim to:

1. Define the core concepts of Comparative Vertebrate Anatomy, both as a scientific field and a teaching unit.
2. Define the core competencies students can gain from the study of Comparative Vertebrate Anatomy.
3. Provide a framework for the assessment, and if needed, refinement of these core concepts and competencies.
4. Outline the Grand Challenges that Comparative Vertebrate Anatomy is facing, in particular with respect to biases, historical and systemic barriers, and their collective impact on our field.

This project was an iterative effort that engaged the Comparative Vertebrate Anatomy community to identify the core concepts, competencies, and challenges of our field. As authors, we aimed to present these with resources that are by no means exhaustive (see also Supplementary data, Table S2) to create a starting point for others to delve deeper either in their own scholarship or teaching. It is our hope that this will become a living document, to be discussed extensively and updated periodically.

### About core concepts and competencies

*Core concepts* are “big ideas” or foundational concepts that are central to a discipline (National Research Council 2007). They should provide coherence or structure to a field while being transferable across sub-disciplines within the field (Michael et al. 2017). AAAS's “Vision and Change” document (2011) states that core concepts “provide a set of overarching principles that are important throughout the living world, and their use in teaching biology lends meaning to the multitude of facts that the students encounter in any undergraduate biology course” (p. 11). *Core competencies* are the skills that students need to develop and apply within a field to “understand, generate, and communicate knowledge about the living world” (AAAS 2011, 11).

Focusing on core concepts and competencies benefits both students and instructors. Core concepts reduce the amount of material to know, since the focus is no longer on memorization of content but on grasping larger ideas; this can foster retention of information and thus deeper comprehension (Chang et al. 2014). This is not to say that learning terminology and some content is not important; one has to learn the language and data available to communicate in the discipline. However, a focus on core concepts and competencies, and an appreciation of the limitations of existing information, means that anatomical systems and representative taxa are used as illustrations of the overarching themes and not as the end point of learning itself. This affords instructors the time and space to draw on their own expertise and affinities, which leads to more enthusiastic presence in the classroom and, in turn, has a positive effect on all students but especially those from non-traditional backgrounds, such as students who transfer from 2-year colleges to 4-year degree-granting institutions (Dorame 2012). Core competencies and concepts are meant to provide a framework for the course, helping new instructors design their own courses but also giving more experienced instructors the opportunity to evaluate and rethink current course structures and pedagogies.

Core concepts provide scaffolding for further learning of new concepts since each concept should be transferable across disciplines (Wiggens and McTighe 2005). Each new system in which the core concepts are applicable should be easier to understand because the student already has a base of knowledge (Michael et al. 2017). Both core concepts and competencies contribute to the development of problem solving skills (AAAS 2011; Michael et al. 2017), a skillset projected to be more important for the future workforce than knowledge in any particular field (NSF 2018).

Learning outcomes are specific applications of the core concepts or competencies (Tansey et al 2013). We give examples of learning outcomes for each core concept. However, we expect that specific learning outcomes will differ among instructors, reflecting the instructor's focus and expertise.

### Process for defining core concepts and competencies

While there are three authors on this paper, we recognized that we needed broad input and agreement from a variety of stakeholders to establish the core concepts and competencies of Comparative Vertebrate Anatomy. Therefore, we followed the best practices of other biological subdisciplines (American Society of Plant Biologists 2017; Michael et al. 2017). We note that Miller et al. (2002) proposed concepts and skills for Anatomy (in the context of Anatomy and Physiology) that were generated by one author and refined via feedback from former students and some American Association of Anatomists members. While there is a little overlap with those presented here, Miller et al.’s concepts and skills include items that fall under the process of learning in general.

In early 2019, we asked members of the SICB’s DVM to contribute their ideas for the core concepts as an interactive poster at the annual meeting (Whitenack et al. 2019) and through a Google Form. Responses were used to compose a first draft of core concepts, which was then shared with a subset of SICB DVM members and via social media in August 2020 for further feedback. As we continued to refine the core concepts, it became clear that skills—or core competencies—were also of importance to the community. We wrote the core competencies based on SICB member feedback, then sent a final draft of both the core concepts and competencies to the SICB DVM community for a final round of feedback in spring 2021. The list of core concepts and competencies presented here is based on the combined results of the extensive feedback we received, our interpretation of the literature, and our own teaching experiences.

## Core concepts

We present five core concepts of Comparative Vertebrate Anatomy (Supplementary data, Table S1) that partially, but not surprisingly, overlap with those defined for Biology overall in Vision and Change (AAAS 2011).

- Evolution: The diversity, variation, and unity of vertebrate anatomy are explained by descent with modification.
- Structure and function: The structure of vertebrate anatomy is under heavy selection to match its functional demands; but because demands change over time and evolution acts on what already exists, an anatomical component at any given time may not have an optimal structure for its current function.
- Morphological development: Vertebrate anatomy is expressed as phenotypes that are the result of genotypes executed through a developmental program. Major shifts in phenotype can be achieved through modularity, which allows certain aspects of the phenotype to undergo major variations yet remain integrated in other ways.
- Integration: Anatomical structures develop, function, and evolve as integrated modules. These processes occur across space, time, and biological levels of organization.
- Human anatomy is a result of vertebrate evolution: As vertebrate animals, human form has been constrained by phylogenetic ancestry.

Each core concept of Comparative Vertebrate Anatomy could generate enough material to fill a semester's worth of lessons, but here we provide a summary of each, with potential learning outcomes and resources for further reading or use in the classroom. Neither the suggested learning outcomes nor potential resources are meant to be exhaustive, but rather are meant to be used as starting points for each instructor to tailor the class to their expertise and level of students. The intrinsic ideas of each concept can be found in Supplementary data 1, Table S1, which also includes elaboration with examples. Additional resources can be found in Supplementary data 2.

**Fig. 1:**
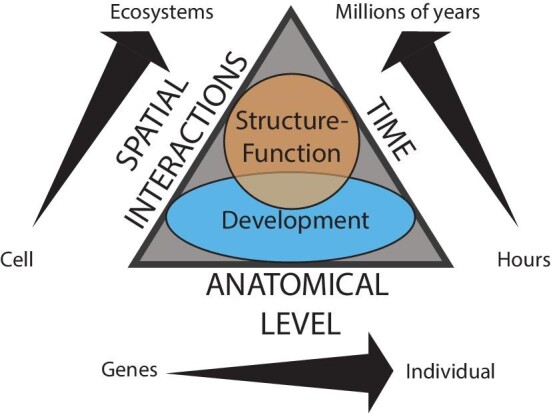
Graphical representation of the core concepts of Comparative Vertebrate Anatomy highlighting the integration of all core concepts. Any given part of vertebrate anatomy is inherently integrative (core concept D) because it is determined by three axes: interactions in space (spatial interactions), time, and level of biological organization (anatomical level). Spatial interactions span cell-to-cell to ecosystem level interactions. Although further spatial interactions are possible, they are likely rare and unlikely to have had an effect on anatomical evolution. Temporal factors range from a few hours, the time it takes for the anatomy to develop embryologically, to millions of years, the amount of time represented in the fossil record. Vertebrate anatomy is the sum of evolutionary modifications at multiple levels of biological organization, termed here anatomical level. Therefore, Comparative Vertebrate Anatomy can be studied anywhere along the continuum of these three axes. The triangle formed by the apices of the three axes represents Evolution (core concept A). Structure-function (core concept B), acts across all anatomical levels to give rise to vertebrate anatomy but within a narrower range of spatial interactions and time. Structure-function for anatomical structures is not possible before a structure is formed; hence, this concept does not encompass individual cell-to-cell interactions. Similarly, at higher complexity spatial interactions each interaction is likely to have a negligible effect on the structure–function relationship of a single anatomical structure. Structure–function is also unlikely to be a significant factor during development because the structure is not yet fully formed. Development (core concept C) is true for all anatomical levels, from genes to individuals, with influence from spatial interactions that fade near the inter-individual level and at the time span of an animal's lifespan. Structure–function and development overlap because structure cannot evolve without developmental pathways changing.

It is also important to note that the borders between individual core concepts are not absolute, but rather the core concepts bleed into each other (Fig. 1). For example, evolution is listed as its own core concept, but is an integral part of every other core concept. Evolutionary processes operate across the three axes of space, time, and anatomical level through development (and genetics) and structure–function relationships.

Therefore, when teaching an intrinsic idea (Supplementary data, Table S1) of the core concept of evolution, an instructor may choose to place emphasis on the fossil record to illustrate the major transformations in vertebrate anatomy, such as from water to land, while another may focus on the developmental pathways that gave rise to the structures that made the transition possible. Such major transformations also open new functional niches allowing for potential rapid speciation and morphological diversification with examples including the origin of jaws, the evolution of powered flight, and the modification of fins into limbs.

### A. Evolution

Vertebrate animal forms are the result of evolution. The most commonly discussed (from our surveys, but also in textbooks) evolutionary concept in relation to Comparative Vertebrate Anatomy is that of homology (Hall 1994; Wagner, 2016), as it illustrates the common ancestry of vertebrate animals and descent with modification throughout lineages.

As instructors explore vertebrate anatomy with students, other concepts that will come up are natural selection, descent with modification, and the notion of evolutionary constraint. Natural selection is a process that acts on an organism's overall phenotype. Therefore, anatomical structures that have the potential to improve an animal's fitness are likely under selection because their functional performance influences survival. For this reason, many animal forms are directly related to their function in their environment (see Core Concept B; structure and function). Critical to the theory of evolution by natural or sexual selection is the fact that anatomical structures vary within a species (Carroll 2005b; Charlesworth et al. 2017). However, some anatomical forms are constrained by evolutionary history or by developmental, structural, or functional integration with other parts. Traits that covary among species over evolutionary time (co-evolve) are considered to be evolutionarily integrated. Therefore, vertebrate phylogenies provide frameworks for the comparison of species’ forms and a deeper understanding of vertebrate anatomy. “Tree thinking” is also defined as a core competency (Core Competency F).

#### Possible learning outcomes

1. Students should be able to explain descent with modification of the axial and appendicular skeleton as vertebrate species transition from living in water to living on land.
2. Students should be able to describe an example of a trait that is homologous for some lineages but homoplasic in other lineages (e.g., powered flight or endothermy).

#### Potential resources

1. Dial KP, Shibin N, Brainerd EL. 2015. Great transformations in vertebrate evolution. Chicago: University of Chicago Press.
2. Stephenson A, Adams JW, Vaccarezza M. 2017. The vertebrate heart: an evolutionary perspective. J Anat 231: 787–97.
3. Brazeau MD, Friedman M. 2015. The origin and early phylogenetic history of jawed vertebrates. Nature 520: 490–7.

### B. Structure and function

One of the most accessible aspects of studying biology is the relationship between the morphology of an organism and its lifestyle. The relationship between structure and function is pervasive throughout biology at all levels of biological organization and is one of the core concepts identified in the AAAS's “Vision and Change” (2011). In the context of Comparative Vertebrate Anatomy, the relationship still spans levels of biological organization—from cells to tissues, organs, body regions, and whole organisms—but is focused on the morphology of anatomical structures. The function of anatomical structures must obey the laws of physics. Therefore, depending on the anatomical systems an instructor chooses to illustrate this principle, the appropriate background in physical laws must be covered. Because structure is often an indication of function, and function is directly correlated with ecology, we expect that morphology will also correlate with ecology, which is where natural selection takes place. The study of the relationship between a given structure and its function at a given point in time is known as functional morphology.

Natural selection on structures for performance only occurs on existing structures; therefore, there are finite solutions to functional demands. Additionally, structures are never selected upon in isolation, but as part of an integrated organism and usually need to fulfill multiple functions, often with competing requirements (trade-offs). This reality often gives rise to structures that, based on physical principles alone, appear to be suboptimal. Natural selection can also give rise to the existence of multiple structural solutions to a single functional demand, either because the functional demand is so critical to organismal fitness that redundancy is a requirement, or because function is not constant and subtle variations in function require structural variation. Therefore, to understand the diversity of vertebrate anatomy over time and space, a comparative approach that includes the other core concepts must be employed (Fig. 1).

#### Possible learning outcomes

1. Students should be able to describe two structural solutions to the same functional demand.
2. Students should be able to explain how a structure is fit for its function, based on the laws of physics.
3. Students should be able to give an example of suboptimal function of a structure as a result of phylogenetic constraints.

#### Potential resources

1. Vogel S. 2003. Comparative biomechanics: life's physical world. Princeton: Princeton University Press. We also recommend any of the books by this author, even though non-vertebrate examples are included.
2. Jones KE, Ruff CB, Goswami A. 2013. Morphology and biomechanics of the pinniped jaw: mandibular evolution without mastication. Anat Rec 296: 1049–63.
3. Leigh SC, Papastamatiou YP, German DP. 2018. Seagrass digestion by a notorious “carnivore.” Proc Roy Soc B 285: 20,181,583.
4. See a list in Supplementary data 2 for resources such as 3D models of structures and computer models of these structures in motion. Contact the authors to submit more resources to be shared and access the live document.

### C. Morphological development

Genotypes code for phenotypes and morphological development implements this genetic plan for traits. Developmental pathways can be varied (e.g., spatial and/or temporal perturbation of gene expression), can act at the cellular, tissue, and structural levels, and can limit direct correlations between genotype and phenotype (Conith et al. 2020). Modifications along these pathways create new morphologies that are selected upon to generate the diversity of vertebrate forms that have existed. Therefore, the way that an embryo develops provides a conceptual basis for understanding the evolutionary history of vertebrate structures (Hall 2003).

A foundational component of morphological development is that traits, genetics, and developmental pathways can be modular (independent) and have varying levels of structural and/or functional integration (covariance). A developmental module can be transferred to a petri dish or another location on the embryo, and will generally continue to develop as a unit (Schlosser and Wagner 2004). Major anatomical changes can be brought about by modifications to a single developmental module, which allows certain aspects of the phenotype to undergo major variations yet remain integrated in other ways (e.g., functionally; see also Core Concept D: Integration). Trait modularity and integration span levels of time (e.g., evolutionary modules) and anatomical organization (Fig. 1).

Developmental modularity can be illustrated for students by examining segmentation, the fundamental organizing principle of most animal bodies, including vertebrates. Each body segment, e.g., a vertebral body or a pharyngeal arch, is a module. Multiple vertebral bodies in a region, e.g., lumbar or thoracic, also form a module together. In some examples, like the mammalian vertebral column, the segments are functionally integrated, constraining major changes in phenotype (e.g., Galis et al. 2014; but see Varela-Lasheras et al. 2011). There are also examples, like the ostariophysan Weberian apparatus, where modules become highly modified structurally (compared to the remaining segments) to accommodate an evolutionarily new integration with the swimbladder and inner ear (e.g., Bird et al. 2020).

#### Possible learning outcomes

1. Students should be able to give an example of a developmental or genetic pathway and how it has been modified to generate diversity in vertebrate form.
2. Students should be able to identify the limitations of mouse and rat developmental studies for human health.

#### Potential resources

1. Carroll SB. 2005a. Endless forms most beautiful: the new science of evo devo and the making of the animal kingdom. New York: Norton.
2. Kampourakis, K. and Minelli, A. 2014. Evolution makes more sense in the light of development. Am Biol Teach 76: 493–8.
3. Fleming A, Kishida MG, Kimmel CB, Keynes RJ. 2015. Building the backbone: the development and evolution of vertebral patterning. Development 142: 1733–44.

### D. Integration

Vertebrate anatomy is the result of processes across space and time that occur at all levels of biological organization (Fig. 1), making the organism a collection of interconnected systems whose understanding cannot be separated from its environment or its evolutionary history. As a result, the study of Comparative Vertebrate Anatomy must integrate evidence from seemingly diverse fields, such as genetics, mechanics, physiology, biochemistry, ecology, and paleontology (Wake 2008). Furthermore, the student of comparative anatomy needs to (1) be well versed in identifying the multiple levels of questions that need to be addressed when analyzing the anatomy of an animal or system (Fig. 1); (2) be able to identify the types of data that would support or refute any given hypothesis that aims to understand the evolution of a particular anatomical structure; and (3) synthesize data from a diversity of technical fields. For example, a complete analysis of the comparative anatomy of the respiratory system will include comparisons among taxa that differ in their environmental adaptations (Fig. 1; Spatial Interactions axis), comparisons with close and distantly related taxa (Fig. 1; Time axis), and an analysis of aspects, such as tissue development and organismal function (Fig. 1; Anatomical Level axis). Recent interactive technologies, such as Prezi, provide excellent opportunities to visually plot some of these dimensions to improve student learning in the classroom (Ortega and Brame 2015).

The concept of integration is important not only because it is a useful learning tool (AAAS 2011) and a skill for students to have once they enter the workforce (Carratozzolo et al. 2021), but also because it can provide insights into biological processes at the genetic, functional, or ecological level. The reverse is also true: by examining biological processes at the genetic, functional, or ecological level, we can obtain insights into the patterns of anatomical evolution. Integration is therefore a fundamental core concept because it reflects true biological processes.

#### Possible learning outcomes

1. Students should be able to consider at least two levels of integration beyond form-function when evaluating the morphology and evolution of an anatomical structure.
2. Students should be able to provide data from multiple fields (e.g., paleontology and developmental biology) to discuss descent with modification of an anatomical system.

#### Potential resources

1. Orkney A, Bjarnason A, Tronrud B, Benson R. 2021. Patterns of skeletal integration in birds reveal that adaptation of element shapes enables coordinated evolution between anatomical modules. Nature Eco Evol 5: 1250–8.
2. Higham T, Ferry L, Schmitz L, Irschick D, Starko S, et al. 2021. Linking ecomechanical models and functional traits to understand phenotypic diversity. Trends Ecol Evol 36: 860–73.
3. Evans KM, Waltz BT, Tagliacollo VA, Sidlauskas BL, Albert JS. 2017. Fluctuations in evolutionary integration allow for big brains and disparate faces. Sci Rep 7: 1–11.
4. Brainerd, EL. 2015. Major transformations in vertebrate breathing mechanisms. In Great transformations in vertebrate evolution. University of Chicago Press, 47–62.

### E. Human anatomy is the result of vertebrate evolution

Human anatomy is a special case in Comparative Vertebrate Anatomy. A major misconception among the general public is that vertebrate evolution is a linear progression from aquatic fish to terrestrial mammals (and humans) at its pinnacle (Omland et al. 2008; Smith 2009). Understanding this core concept will be beneficial to all students, in their professional and personal lives as patients and stewards of the natural world.

In health-related fields, the misconception that humans are unique among vertebrates leads to medical thinking that is often focused on single systems. Yet the principles of integration apply to humans as well. Recent advances in biomedical science, for example, have established the gut-brain axis (Stilling et al. 2014; Cryan et al. 2019), and conditions such as endometriosis that affect multiple organ systems are treated as isolated gynecological dysfunctions when an integrative comparative approach would have sped therapeutic discovery and improved the lives of millions.

Understanding the phylogenetic placement and evolutionary history of humans within all vertebrates yields an understanding of human anatomy in general. For example, in humans, the left recurrent laryngeal nerve takes a longer, less efficient route than it does on the right side, due to the evolutionary history of the aortic arches (see Shubin 2008). In the context of Comparative Vertebrate Anatomy, this seemingly strange structure makes sense. Understanding phylogenetic history also explains pathologies such as the presence of branchial arch fistulas in some infants due to incomplete closing of pharyngeal pouches or the higher occurrence of inguinal hernias in people with external testes compared to those with internal ovaries. Additionally, appreciating the phylogenetic position of humans in the evolution of all vertebrates will afford our students a better understanding of the utility of model organisms in the study of human health and disease.

A separate benefit of studying human anatomy as a special case of Comparative Vertebrate Anatomy is an appreciation for the diversity of life and the lessons it can teach us. If we understand that the structural design and functional performance of some human anatomical parts are less than optimal because of phylogenetic constraints and/or functional compromises, then we are more likely to seek insights into structure–function relationships elsewhere and, we hope, more likely to appreciate and be moved to protect biodiversity.

#### Possible learning outcomes

1. Be able to describe how the axial musculature of tetrapods is modified to support bipedality in humans.
2. Explain an example of a suboptimal structure–function relationship in humans that is a result of vertebrate ancestry.

#### Potential resources

1. Shubin N. 2008. Your inner fish: a journey into the 3.5-billion-year history of the human body. New York: Vintage Books.
2. Venkadesan M, Yawar A, Eng CM, Dias MA, Singh DK, Tommasini SM, Haims AH, Bandi MM, Mandre S. 2020. Stiffness of the human foot and evolution of the transverse arch. Nature 579: 97–100.
3. Danowitz M, Solounias N. 2016. Embryology, comparative anatomy, and congenital malformations of the gastrointestinal tract. Edorium J Anat Embryol 3: 39–50.

## Competencies

Based on the responses to our surveys, it is clear that experts in the field also emphasize core skills or competencies in their instruction. Many of these competencies overlap with and augment the comprehension of the defined concepts and overlap with those of other fields (AAAS 2011; Hilborn and Friedlander 2013; Clemmons et al. 2020).

The intrinsic ideas of each competency can be found in Supplementary data 1, Table S2, which also includes elaboration with examples. Additional resources, including activities and primary literature, can also be found in Supplementary data 2. As with the core concepts above, the skills an instructor chooses to emphasize in a particular course may depend on available time, materials, or course level. For example, if students do not have a foundational understanding of phylogenetic trees, then an instructor may choose to spend more time on this skill to ensure students are competent. The core competencies described here are as follows:

- Tree thinking;
- Observation;
- Dissection of specimens;
- Depiction of anatomy;
- Appreciation of the importance of natural history collections;
- Scientific communication;
- Data integration.

### A. Ability to apply tree thinking to the study of comparative vertebrate anatomy

Phylogenetic trees are often used in textbooks to illustrate speciation, biodiversity, and the mechanics and patterns of evolution (Catley and Novick 2008—referred to in Eddy et al. 2013). In Comparative Vertebrate Anatomy textbooks, for example, phylogenetic trees are used to illustrate relationships between taxonomic groups (Liem et al. 2001; Kardong 2018). Phylogenies allow for the reconstruction of hypotheses of the evolutionary history of those groups, showing plesiomorphic (ancestral) and apomorphic (derived) traits that unite those taxa to understand descent with modification of a feature.

Phylogenetic trees are also hypotheses about evolutionary relationships and processes, as the distribution of taxa and the topology of the tree are determined by the characters and models chosen to build the tree (Haber 2005). When models are changed or new data are added, tree topologies can change. The evolution of the Chelonia (turtles) is one such example. In addition to giving insights into the evolution of unique features, such as the placement of the scapula within the thoracic cavity in Chelonia, this skill will also inform the process of anatomical evolution for widely inherited traits, such as bone and cartilage. Students can only appreciate the uniqueness of Chondrichthyes if they can interpret from a phylogeny that extinct bony fish existed long before the appearance of Chondrichthyes. Thus, students of Vertebrate Anatomy should have a basic understanding of established relationships of vertebrate lineages while also acknowledging that new data are added to phylogenetic hypotheses regularly and may change those relationships.

However, “tree-thinking” (*sensu*Baum et al. 2005) is a challenging skill to master (e.g., Baum et al. 2005; Gregory 2009 and references therein; Meisel 2010 and references therein), and common misconceptions can persist well beyond the undergraduate level (Crisp and Cook 2005; Sandvik 2008). For example, one common misconception in Vertebrate Anatomy is that evolution is linear (Kummer et al. 2016); understanding how to read branching patterns and the meaning of nodes may help dispel this misunderstanding. Eddy et al. (2013) show that participating in activities that require building trees is one way of helping students perform better on a tree-thinking assessment than a comparison group of students that analyzed existing trees.

#### *Potential learning outcomes* (from Eddy et al. 2013)

1. Students should be able to recognize that traits do not necessarily evolve in a linear manner.
2. Students should be able to recognize that a species cannot be considered higher or lower than others (tree- versus progressivist/ladder-of-life thinking).
3. Students should be able to recognize that extant traits can be considered basal, but that extant species cannot.

#### Potential resources

1. Baum DA, Smith SD, Donovan SS. 2005. The tree-thinking challenge. Science 310: 979–80.
2. Whitenack LB, Drew JA. 2019. Untangling the contribution of characters to evolutionary relationships: a case study using fossils, morphology, and genes. J Biol Educ 53: 217–24.
3. Eddy SL, Crowe AJ, Wenderoth MP, et al. 2013. How should we teach tree-thinking? An experimental test of two hypotheses. Evo Edu Outreach 6: 13.

### B. Ability to apply the skills of observation to the study of anatomical form

The first step to learning Anatomy is to observe anatomical form. Students may “look” at a structure, but will likely need additional guidance on how to actively discern details and how to record these descriptions. As the first step of the scientific method, the formal practice of observation is a paramount skill for any scientist.

In Morphology, the details matter. The ability to differentiate among vertebrate long bones requires an understanding of the various processes, fossae, condyles, and other detailed structures unique to each bone. A small difference in structure, such as the location of a muscular insertion, can result in large differences in function. Careful observation is also the first step in communicating Vertebrate Anatomy. The recording of rich details is necessary for effectively conveying anatomical science among individuals, disciplines, and over time. One method of communicating anatomical science is through anatomical illustrations/depictions, and observation is again the first step toward preparing effective anatomical depictions. In some cases, such observations may even lead to a brand new hypothesis.

#### Potential learning outcomes

1. Students should be able to apply anatomical language, verbally or in writing, to compare the morphology of two or more specimens.
2. Students should be able to identify the taxon to which a specimen belongs, based on careful anatomical observation.

#### Potential resources

1. Nordquist R. 2019. Classic essay on observation: “Look at Your Fish!” ThoughtCo. (https://www.thoughtco.com/look-at-your-fish-by-scudder-1690049).
2. Bixler A. 2016. It's a crocodile! No, a fish! No, a Dolphin! Interpreting evolutionary history from fossil evidence. National Center for Case Study Teaching in Science. (https://sciencecases.lib.buffalo.edu/collection/detail.html?case_id=869&id=869).
3. Moore CM, Lowe C, Lawrence J, Borchers P. 2011. Developing observational skills and knowledge of anatomical relationships in an art and anatomy workshop using plastinated specimens. Anat Sci Educ 4: 294–301.

### C. Ability to effectively dissect specimens

Dissection is the classic laboratory activity in Vertebrate Anatomy. Dissection is a tactile skill that is not only important for future clinicians but also for future technicians in the biotechnology industry since many processes for device production involve the harvesting of organs or cells from necropsies of animals or humans.

The advent of technological tools for anatomical education poses the question: Is dissection a necessary skill? There has been robust discussion regarding human dissection and Gross Anatomy (e.g., Elizondo-Omaña et al. 2005; Flack and Nicholson 2018; Ross et al. 2020; Saverino 2020), with very little regarding Comparative Vertebrate Anatomy (but see Vartak et al. 2019). We argue that since tissues respond differently to forces, it is important for students to develop tactile knowledge of vertebrate tissues. Since a photographic atlas of anatomy, a computer screen, or a virtual dissection table are all two-dimensional (2D), there are limitations on learning the three-dimensionality and integration of the vertebrate body. Furthermore, interindividual variation in anatomy can only be experienced by the dissection and observation of many specimens and is critical not only for appreciating the process of evolution but also for a deeper understanding of the human body for pre-health career students.

A foundational component of the skill of dissection is the preservation of structural integrity, which in turn should facilitate the memorization of anatomy. For example, during dissection of skeletal muscles, superficial muscles are bisected and reflected, thereby maintaining the position of each muscle in the specimen and giving students an opportunity to return to the specimen to study. Furthermore, we see specimen dissection as another opportunity to employ active learning in the classroom, through activities such as peer-to-peer teaching (Danos 2019).

#### Potential learning outcomes

1. Students should be able to safely use dissection tools.
2. Students should be able to apply the appropriate tools and techniques for the dissection of different anatomical systems.
3. Students should be able to use a dissected specimen to demonstrate the anatomy of a system to their peers.

#### Potential resources

1. Dissection 101: Dissection Resources for Classroom Use. n.d. PBS LearningMedia. (https://www.pbslearningmedia.org/collection/dissection-videos-for-classroom-use/).
2. Heithaus P. 1999. Kenyon College Cat Anatomy Tutorial. (https://biology.kenyon.edu/heithausp/cat-tutorial/welcome.htm).
3. See Supplementary data 2 for more resources, including online dissection guides.

### D. Ability to depict anatomy

After an anatomist reveals, identifies, and observes anatomical forms, the rendering of observations provides information for broad access and future study. This depiction can be in the form of 2D or three- dimensional (3D) images, (physical or digital) fabrications, schematics, or models along with descriptions of the structural observations. For a student (novice) of Anatomy, the act of depicting anatomy, such as factual drawing, has been shown to improve factual, inferential, and transfer learning of anatomy (Fernandes et al 2018; Cromley et al. 2020). Students do not need artistic training to make a sketch of anatomy because the focus of an anatomical drawing is the important morphological details not the aesthetics of a rendering. For example, the names of blood vessels between branching points are constant, but the path between those branching points can be plastic between individuals (e.g., arteries in the digestive system originate from the aorta and venous drainage collects in the hepatic portal vein but branching to/from individual organs may vary slightly). A simple pathway diagram can eliminate confusion. Simplified schematics that are labeled clearly may convey more anatomical information and may be less confusing than a photograph.

Increasingly accessible 3D images are now available for teaching and learning vertebrate morphology. The same 3D medical imaging techniques, such as computed tomography (CT) scanning and magnetic resonance imaging, that are used for humans are now widely used for vertebrate morphology (Buser et al. 2020). These detailed scans can lead to insights into the anatomy and function of delicate and small structures, especially in small species. Because these digital files are freely available in online repositories, an increasing number of instructors use them to print 3D models of anatomical structures for use as study specimens or for the study of form–function relationships. Annotating and assembling these 3D models is another means of depicting anatomy.

#### Potential learning outcomes

1. Students should be able to draw a simple diagram that illustrates the relative shape and position of components of an anatomical system, e.g., aortic arches.
2. Students should be able to annotate an illustration of an anatomical system.
3. Students should be able to draw simple diagrams to compare the same anatomical system in two or more taxa.

#### Potential resources

1. Fernandes MA, Wammes JD, Meade ME. 2018. The surprisingly powerful influence of drawing on memory. Curr Direct Psychol Sci 27: 302–8.
2. Ainsworth S, Prain V, Tytler R. 2011. Drawing to learn in science. Science 333: 1096–7.
3. Staab KL. 2021. Implementing fabrication as a pedagogical tool in vertebrate anatomy courses: motivation, inclusion, and lessons. Integr Compar Biol 61: 1013–27.

### E. An appreciation of the importance of natural history collections

The study of morphological evolution requires data that spans space and time. Collections curate such data and make it available to students and scientists, speeding up the pace of scientific discovery. Vertebrate anatomists learn and contribute to their field by making use of specimen collections.

Not every Vertebrate Anatomy classroom is located at a university with a curated natural history museum, but most schools do have a classroom skeletal collection of commonly studied species or local species. Furthermore, natural history museums around the world are making their collections available on various online platforms (e.g., oVert funded by the National Science Foundation) and providing a rich resource for students, teachers, and even science enthusiasts. The 3D digital models that are freely shared through resources like Morphosource.org and Sketchfab.com make it possible to 3D print rare specimens at a relatively low cost.

Depending on the course goals, instructors may place different amounts of emphasis on this competency. A more advanced course may prepare students for graduate-level research projects by making use of the physical specimens housed in natural history museum collections. But even an introductory course can expose students to the value of specimen collections so that they can appreciate how progress is made in Comparative Vertebrate Anatomy. Student projects can include taking measurements or making identifications (e.g., ecomorph, color, specimen sex) and returning these data to be associated with the specimens, thereby contributing to improving the quality of the specimens and their associated metadata.

The utility of natural history specimens is greatly improved when accompanied by natural history observations. These observations provide insights into the function and ecology of anatomical structures and hence are the first step toward controlled studies to get to the core concepts of each anatomical structure.

Lastly, anatomical collections, including natural history collections, can serve as bridges between art and science. Such interactions are critical for broadening the impact of research done at these institutions and potentially generating interest in STEM among students that would otherwise not be exposed to this field (Sysling 2016; see “Picturing Science” show http://www.picturingscience.com/about-cleared).

#### Potential learning outcomes

1. Students should be familiar with the types of material and data that are housed in natural history collections.
2. Students should be able to form hypotheses based on data from a natural history collection.

#### Potential resources

1. Colella JP, Bates J, Burneo SF, Camacho MA, Carrion Bonilla C, Constable I, D'Elía G, Dunnum JL, Greiman S, Hoberg EP, Lessa E. 2021. Leveraging natural history biorepositories as a global, decentralized, pathogen surveillance network. PLoS Pathogens 17: e1009583.
2. Buckner JC, Sanders RC, Faircloth BC, Chakrabarty P. 2021. Science forum: the critical importance of vouchers in genomics. Elife 10: e68264.
3. Resources in Supplementary data 2.
4. Howard Hughes Medical Institute. N.d. HHMI BioInteractive: Sorting finch species. (https://www.biointeractive.org/classroom-resources/sorting-finch-species).

### F. The ability to communicate scientific information to peers and the public

Every scientist-in-training must gain practice with sharing knowledge because science is collaborative. The ability to effectively communicate science is a competency highlighted in Vision and Change (AAAS 2011). By practicing how to communicate complex hypotheses about the origin and evolution of vertebrate morphology, students not only learn the material better, but will be better equipped to share their knowledge in public discourse.

Effective scientific communication is especially important because there are many misconceptions and even mistrust among the general public about accepted scientific principles (National Academies of Sciences 2017), including the evolution of vertebrates (Lombrozo et al. 2008). Vertebrate anatomical systems are familiar to most people because many are shared with humans; hence, they could be effectively used to communicate both our best understanding of the process of evolution and the process of scientific discovery in the field.

Additionally, effective scientific communication can generate interest in STEM among students and the public (Whitenack et al. 2021). This interest is necessary because it brings diverse voices, perspectives, and stakeholders to the endeavor of scientific discovery increasing its success and impact (NSF 2018). In fact, new discoveries are the only way in which major breakthroughs in applied science occur (e.g., Ishino et al. 2018; Salata et al. 2019).

#### Potential learning outcomes

1. Students should be able to succinctly summarize a scientific paper/discovery/knowledge about the evolution of an anatomical system, using a combination of scientific and lay language, in a way that can be understood by the general public.
2. Students should be aware of the quality of science communication outlets that exist and be able to distinguish between reliable and unreliable sources.
3. Students should actively engage in science communication within their organization.

#### Potential resources

1. Zimmer C. N.d. Science writing: guidelines and guidance. (https://carlzimmer.com/science-writing-guidelines-and-guidance/).
2. Patek S. 2016. Why knowledge for the pure sake of knowing is good enough to justify scientific research. PBS NewsHour. (https://www.pbs.org/newshour/show/why-knowledge-for-the-pure-sake-of-knowing-is-good-enough-to-justify-scientific-research).
3. Pollett S, Rivers C. 2020. Social media and the new world of scientific communication during the COVID-19 pandemic. Clin Infect Dis 71:2184–6.
4. Kelly D. 2012. What we don't know about penis anatomy. TEDMED. (https://www.ted.com/talks/diane_kelly_what_we_didn_t_know_about_penis_anatomy).

### G. The ability to integrate data from multiple subdisciplines, applied to vertebrate anatomy

Evolutionary biology, of which Comparative Vertebrate Anatomy is a subfield, is a historical science (Smocovitis et al. 1992). As a result, evolutionary biologists often act as detectives to reconstruct the evolution of anatomy, using evidence from multiple sources, all of which must agree based on the fundamental principles of biology, physics, and chemistry (Wake 2003). Understanding how to read and interpret evidence from embryology, paleontology, biomechanics, and comparative biology is necessary for evaluating hypotheses regarding morphological evolution. Hence, data integration is a core competency for any student of evolutionary biology.

Conversely, integration of evidence from multiple sources provides clues as to the fundamentals of biological and evolutionary processes (Wake 2003). Comparative Vertebrate Anatomy can be a framework for interpreting and mining the exponentially increasing collection of genetic data (Koentges 2008). Since life is inherently integrative (see Fig. 1), as we try to understand more complex systems, integrating data from multiple fields will be a critical skill (Krumholtz 2014; Chandrasekaran et al. 2021). In the age of big data, critically evaluating multiple sources of information will be a fundamental skill for any worker, especially in biology.

Inherent in the skill of integrating data are effective collaboration with multiple experts from different subfields (Wake 2003). Scientists with diverse expertise, including those in non-biological fields, can collaborate and integrate data for a synthetic approach to the research and teaching of Comparative Vertebrate Anatomy and Morphology. We argue that any field of science is strengthened with more diverse perspectives and approaches.

#### Potential learning outcomes

1. Students should be able to cite multiple sources of data when explaining the major evolutionary transition from water to land in tetrapods.
2. Students should be able to use both paleontological and neontological sources of data to explain the transition from fish jaw to mammalian inner ear bones.

#### Potential resources

1. Da Silva FO, Fabre AC, Savriama Y, Ollonen J, Mahlow, K, Herrel A, Müller J, Di-Poï N. 2018. The ecological origins of snakes as revealed by skull evolution. Nature Commun 9: 1–11.
2. Dial KP, Shibin N, Brainerd EL. 2015. Great transformations in vertebrate evolution. Chicago: University of Chicago Press.
3. Koentges G. 2008. Evolution of anatomy and gene control. Nature 451: 658–63.

## Grand challenge—inclusivity, diversity, and decolonization in Comparative Vertebrate Anatomy

As we worked with the contributions of our colleagues to establish these core concepts and competencies, we were struck by how the human element of Comparative Vertebrate Anatomy was mentioned by only a few respondents. This was not surprising; the introductory chapters of many Comparative Vertebrate Anatomy textbooks give a fairly brief overview of the history of the field (e.g., Kent and Carr 2001; Kardong 2018). Textbooks also provide a list of references at the end of the chapter but often do not talk about the scientists within the chapter (Kent and Carr 2001; Liem et al. 2001). What we find to be missing is consideration of the people in this field beyond a list of names: the diversity of people who have and currently work in our field, the honest history of those people, and how their scientific pursuits have affected others. Science is not as objective as we like to think or hope, and Comparative Vertebrate Anatomy is no exception.

As we think about the future of our field—our students—it is imperative to create a welcoming community for everyone. Currently, both students and faculty of color are underrepresented in biology at large. For example, from 2014 to 2018, African Americans earned approximately 1% and Alaskan Natives or American Indians earned 0.2% of PhDs in ecology and evolutionary biology (Tseng et al. 2020). Systemic racism in STEM, the collection of practices that on purpose or inadvertently make success harder for a racial group, deters historically excluded people from remaining in our field; this begins in K-12 and persists beyond earning a PhD (e.g., Young 2005; Morales et al. 2020; O'Brien et al. 2020; Singleton et al. 2021 re: ecological field experiences). Gender essentialism, the belief that gender categories are biological and discrete (often conflating sex and gender), and gender stereotypes also contribute to gender disparities in STEM fields (e.g., Cecsi et al. 2009; Nosek et al. 2009; Cooper and Brownell 2016; Donovan et al. 2019).

Addressing diversity explicitly in the classroom and incorporating anti-racist pedagogy increases retention of underrepresented students (e.g., Hagedorn et al. 2007; Johnson 2007; Cronin 2021); retaining students is necessary for the future of Comparative Vertebrate Anatomy. For example, cultivating a sense of belonging, which includes exposure to same-race and same-gender role models, is correlated with an interest in graduate school in ecology and evolutionary biology (Marx and Roman 2002; Dasgupta 2011; Graves 2019; O'Brien et al. 2020). Numerous studies have also shown that college science classrooms are interpreted as white spaces and not neutral with regard to gender and race (Tanner and Allen 2007; Archer et al. 2015; Ryu 2015; O'Brien et al. 2020). Anti-racist pedagogical practices can help foster inclusion by reducing or removing barriers, reducing academic inequities, and helping to create a culture of anti-racism (Cronin et al. 2021).

Many of our students will not go on to become vertebrate morphologists. However, we know that biology content in textbooks and the classroom influences individual beliefs associated with gender and racial disparities (e.g., Donovan 2017; 2019). Many Comparative Vertebrate Anatomy students’ career goals are in the field of human medicine. In the USA, both individual and structural racisms (racism supported by laws, rules, cultural and societal norms, and economic systems [Bailey et al. 2017; Rothstein 2017]) have direct ties to health inequities (Bailey et al. 2017; 2021 and references therein; Sequist 2017 and references therein). Addressing racism, sexism, and other -isms head-on helps disrupt these problematic beliefs, disparities, and systems.

As educators, doing something about this starts with creating a classroom environment where *each* student can see themselves, where *each* student feels valued, and we acknowledge how our field has impacted people for better *and* for worse. We have chosen to discuss these topics under the heading of “grand challenges” rather than “core concepts” because our field is still in the first steps of naming and acknowledging these important issues. Below, we highlight some areas where we, as a community, can begin our discussion of how to move forward.

### The giants of our field

The traditional telling of the history of Vertebrate Anatomy and Morphology in general is a Western one: Ernst Haeckel, Herbert Spencer, Thomas Henry Huxley, Carolus Linnaeus, Johann Wolfgang von Goethe, Pierre Belon, George Cuvier, Edward Drinker Cope, Charles Darwin, Alfred Russel Wallace, Louis Agassiz, and so forth. Moving forward in time to the 20th century focuses mostly on the USA and European contributions to the field: Ernst Mayr, Stephen Jay Gould, Carl Gans, R. Glenn Northcutt, Karel Liem, David Raup, Marvalee Wake, and Pieter Dullemeijer to name a few (e.g., Hall 2005).

If we include the field of Human Anatomy, as we argue here as being a special case of Vertebrate Anatomy and Morphology, then the timeline is often extended back to Aristotle (384–322 BCE), Herophilos (335–280 BCE), and Galen (129–216 CE). However, we know that there were absolutely contributions from other regions of the world. Egyptians as early as 4000 BCE engaged in anatomical study and documentation as they practiced surgery and mummification; any mentions of their contributions tend to be brief if present at all (e.g., Kent and Carr 2001). Notable examples include the Ebers Papyrus (1550 BCE), which details the human cardiovascular system, and the Kahun Papyrus (1850 BCE), which correctly links the placenta to fetal nourishment (Tubbs et al. 2019). Anatomical hieroglyphs reflect knowledge taken from animal slaughter and mummification, particularly bovines (Schwabe et al. 1982). The Indian physician Sushruta, who is known as the Father of Indian Surgery and is estimated to have lived sometime between 1000 BCE and 500 CE (Tipton 2008), offered insights into muscles and joints via human dissection (Tubbs et al. 2019). After Aristotle and Galen, anatomical discoveries continued outside of the Western world, despite the stories in our textbooks. For example, in the 13th century CE, an Arab physician named Ibn al-Nafis was the first to correctly posit the separation of blood in the right and left ventricles of the heart, to determine the path of pulmonary circulation, and to predict the presence of pulmonary capillaries; the latter predated this same discovery in Europe by 300 years (West 2008). Non-Western ways of knowing are starting to be better incorporated into other natural science fields and their classrooms (e.g., Kimmerer 2002; Lemus et al. 2014); however, they have not yet been integrated into Comparative Vertebrate Anatomy. For example, one may consider acupuncture, a technique based on traditional Chinese medicine and used by Western medicine for a number of ailments. While researchers have not pinpointed the exact mechanisms through which acupuncture acts, this could be incorporated into a discussion of peripheral nervous system anatomy or neurophysiology. We suggest Brenna's (2022) paper for an excellent summary of the history of human anatomical study that includes non-Western history.

We also note that women are typically left out of the narrative as well. While the English paleontologist Mary Anning is known by many, other female anatomists such as Anna Morandi (18th century CE, Italy, specialized in sensory organs) and Marie Marguerite Bihéron (France, 1719–1795, made extremely detailed wax models of human anatomy) are likely unknown to many of us. Similarly, the contributions of Black scholars are often left out of the narrative. For example, W. Montague Cobb (1904–1990) was a prolific scholar whose work included Anatomy and the effects of racism on science; he used scientific techniques to counter the assertions by eugenic scientists (see “Comparative vertebrate morphology and racism”). Yet he is largely unknown to white scholars (Blakey and Watkins 2022). We highly recommend the recent paper by Blakey and Watkins (2022) for a discussion of Cobb's work and legacy.

By limiting the foundational history of Comparative Vertebrate Anatomy to only being inclusive of white men, we are not only inaccurately portraying the history of our field, but we are also (either intentionally or unintentionally) signaling to our students that one has to be a white man to be a successful scientist (Good et al. 2010; Wood et al. 2020). Highlighting past and current work by scientists that is diverse and relatable to our students can help bolster science identity (Schinske et al. 2016; Wood et al. 2020; Cronin et al. 2021) and minimize stereotype threat (e.g., Steele and Aronson 1995; McIntyre et al. 2004), both of which, in turn, contribute to persistence in STEM (Seymour and Hewitt 1997; Brickhouse et al. 2000). Highlighting scientists that reflect the diversity in our field is an easy first step, and activities such as Scientist Spotlight are an appropriate way to do that (e.g., Schinske et al. 2016, Yonas et al. 2020). However, if this is the only step that is taken, those same individuals that we spotlight become tokenized, and meaningful change is not made.

### The contributions of colonialism and slavery to our field

There can be no Comparative Vertebrate Anatomy without specimens to study. The collections of Western natural history museums, some of which originated from those same pioneers that we read about in our anatomy textbooks, have problematic origins steeped in violence and erase the non-European people who were involved in their collection. For example, narratives about Charles Darwin's famous voyage on the *HMS Beagle* focus on the specimens he returned with and how the trip led to his proposal of natural selection. However, these narratives tend to leave out the fact that one of the expedition's goals was to facilitate British control of the Falklands, Galapagos Islands, and South American coastline or that a Guyanese emancipated enslaved man named John Edmonstone taught Darwin the taxidermy skills he used to preserve his finches (Desmond and Moore 2009; Das and Lowe 2018). Alfred Russel Wallace worked with a Malay man named Ali; Ali collected a substantial portion of Wallace's specimens, preserved the skins of birds, and acted as Wallace's guide (Das and Lowe 2018). More specific to Comparative Vertebrate Anatomy, the European “discovery” of the platypus *Ornithorunchus anatinus* is a case study in the erasure of non-white contributors to our field. John Hunter, the second governor of the British colony of New South Wales (Australia), was credited with discovering the platypus, when in fact an Indigenous Australian man was the one who caught the specimen (Home 1802, as cited in Ashby and Machin 2021).

The devastating effects of colonialism, violence, and the slave trade cannot be disentangled from natural history collections (Wynn-Grant 2019). Darwin's expedition was not the only one to include trained soldiers and weapons with the purpose of controlling tracts of land and their inhabitants. Captain James Cook and Napoleon Bonaparte mixed scientific collection with violence and oppression of Indigenous peoples of the places they collected (New Zealand and Egypt, respectively), while Captain Gerald Barrett-Hamilton collected vertebrate specimens at an active concentration camp during the Second Boer War (1899–1902) (specimens now housed at the British Museum of Natural History and the University Museum of Zoology, Cambridge) (Das & Lowe 2018; Ashby and Machin 2021). Sir Hans Sloane's collection trips were funded by profits from the Transatlantic slave trade; the specimens he collected were deposited at the National History Museum and British Museum (Das and Lowe 2018; Delbourgo 2018). To be clear, these men were complicit in the subjugation and destruction of millions of individuals, families, and communities. Unethically acquired human remains, particularly those of Indigenous, African, and African American individuals, are direct examples of colonialism and the slave trade expanding natural history collections; we recommend Williams and Ross (2022) for a discussion of African and African American remains in US natural history collections.

While it is easy for us to focus on the 17th through 19th centuries CE and European countries, it is important to note that these practices continued and that the USA was also a participant. In the current century, we still see the practice of “parachute science”, where scientists travel to another country for field research and then return back to their home country without involving anyone from that country in the subsequent scientific work (e.g., Costello and Zumla 2000; de Vos 2020; North et al. 2020). For example, vertebrate fossils such as the remains of dinosaurs and mammals have been taken from their countries of origin, then studied and deposited in collections elsewhere with no mention or collaboration of local people who participated in the work. In some cases, countries of origin are calling for the repatriation of fossils that were taken without permission or possibly illegally (Vogel 2019; Elbein 2021).

The effects of colonialism and slavery persist in STEM today, from the content of textbooks we use to the measures for academic performance to the structure of academic institutions. Money from the Transatlantic slave trade and the subjugation of Native Americans and their lands helped pay for the founding of many US academic institutions (Harris et al. 2019). Thus, academia has had ties to systemic racism since its establishment in the USA. Graves (2019) details the history of chattel slavery's lasting effects on the diversity of evolutionary biologists today, which likely applies to Vertebrate Morphology as well.

### Comparative vertebrate morphology and racism

Evolutionary Biology has long been used to support racism (see Graves 2015 for a summary). The contribution of Comparative Vertebrate Anatomy to racism is not immediately obvious until we remember that human anatomy is a specialized case of vertebrate anatomy (Core Concept E) and that the same techniques that we use for comparing species have also been applied to comparing human specimens. Western scientists were intrigued by the natural variation in human skulls and skeletons, leading to the acquisition of human remains from all over the world for study in European and North American institutions (Sysling 2016). Several scientists used cranial capacity to estimate intellectual ability, which in turn was used to support both eugenics and sexism, including Karl Pearson, Francis Galton, and Alice Lee. It is noteworthy that while Lee used her studies of cranial capacity to discredit the idea that women are less intelligent than men, she failed to do so for race (McNeill 2019). One of the most influential collections in the USA belonged to physician and scientist Samuel George Morton; we briefly summarize his history here, but see Menand (2001) for more information. Morton used his collection of over 600 skulls to then rank five races in 1849, placing Caucasians on the top of a hierarchy and Native Americans and Black people on the bottom, and using these data argued that the five races were different species. Louis Agassiz visited Morton in 1846 and was so impressed with his work and collection that he became a vocal proponent of continued restriction of the rights of Black people because he believed they were different species (see Menand 2001 for direct quotes from Agassiz). We also note that many of Morton's skulls were acquired via graverobbing, and over 50 are thought to be from enslaved Africans (Kelleher 2021; Williams and Ross 2022).

Agassiz was not the only giant of our field involved in the mix of science and racism. George Cuvier agreed with Louis Agassiz with regard to the hierarchy of human races (Graves 2015). Ernst Haeckel, in an attempt to show that humans evolved from other organisms, also divided humans into different species and arranged them hierarchically (1868, as cited in Levit and Hossfeld 2020). Edward Drinker Cope also elevated white people above others in terms of “intellectual power” (Cope 1887). David Starr Jordan, ichthyologist and founding president of Stanford University, was the chair of the Eugenics Committee of the American Breeders’ Association (Jackson and Weidman 2005/2006) and wrote the essay *The Human Harvest* (1899 and 1907), which argued for racial segregation and purity.

Even though these scientists are long dead, their legacy of racism lives on. Works such as Herrnstein and Murray's 1994 book *The Bell Curve* and Wade's 2014 book *A Troublesome Inheritance: Genes, Race, and Human History* wrongly assert that attributes such as intellect and knowledge are racially and genetically determined (Donovan 2017; Pressman 2017). These are presented under the guise of “science” to support racism (Saini 2019), and while works such as *The Bell Curve* have been largely dismissed, sales of this book increased again in 2017 (Siegel 2017). Studies have shown that there is a causal link between the ways genetics research is reported in journalistic publications and beliefs that genetic differences underlie race (e.g., Phelan et al. 2013; Morin-Chasse 2014). There is no reason to assume that morphology could not be used similarly as it has been in the past. Addressing in the classroom how science impacts society and ethics may help counter this. Liscum and Garcia (2022) outline how they incorporate the history of science and eugenics, centered around genetic engineering, into one of their courses; a similar approach could be taken using Morton's discredited studies as a starting point.

### Heteronormativity, androcentrism, and reproductive anatomy

One area of Comparative Vertebrate Anatomy deserves particular attention: reproductive/urogenital anatomy. Throughout biology as a discipline, we tend to focus on heteronormative reproductive biology in the classroom. This can be challenging for those that identify as LGBTQ+, as they may not feel welcomed or represented if we are teaching reproductive biology along strict binary, heteronormative lines; according to a 2021 Gallup poll of US adults, 15.9% of adults belonging to Generation Z (born 1997–2002) identify as LGBT (Jones 2021). However, even among vertebrates, we know that reproductive biology is more complex than “male” versus “female”; yet we still teach this as binary.

Cooper et al. (2020), Štrkalj and Pather (2021), and Easterling and Byram (2022) give a wonderful set of recommendations for making academic Biology and Anatomy more inclusive for LGBTQ + individuals. In particular, Cooper et al. (2020) and Easterling and Byram (2022) recommend that instructors dissociate the language of gender identity (e.g., boy and girl) from those of anatomy (e.g., penis and vagina). They also recommend that instructors highlight that the same natural variation we see in other traits is also present in sexual characteristics. Štrkalj and Pather (2021) advocate for explicitly teaching “the continuum in between the ‘typical male’ and ‘typical female’ presentations” (Table 1 in Štrkalj and Pather 2021). Given that Comparative Vertebrate Anatomy laboratories are often excellent places for students to engage with natural variation in anatomical structures (e.g., differing meandering paths of blood vessels among individuals), drawing attention to the variation in reproductive structures could help normalize the variation we see in human reproductive anatomy.

Finally, it is important to note that just as anatomical studies of skulls were used to support racism and eugenics, anatomical studies have also contributed to the repression and harm of those people who are LGBTQ+. Smith (2022) provides a thorough summary of queer history and Human Anatomy; here we highlight some of his points. Beginning in the 19th century, comparative studies of genitalia were used to classify lesbians, Asian women, and African women as peculiar or abnormal (Somerville 1994; Dreger 1998). Anal morphology was used to identify and harm homosexual men and is still used in some parts of the world today (Brady 2014; Smith 2022). Intersex individuals and those with ambigous genitalia have been labeled as abnormal and subjected to medical “treatments”, often without their consent (see examples in Smith 2022).

Additionally, researchers have long noticed that there is a male-bias in biomedical and basic research studies (Klein et al. 2015; Clayton 2018) and textbooks (Lawrence and Bendixen 1992; Morgan et al. 2016; Parker et al. 2017). A similar male bias is found in Evolutionary and Comparative Anatomy; for example, the last detailed morphological description of the human mammary gland was done in 1840 by Sir Astley Paston Cooper (Copper 1840). Comparative biologists are beginning to fill in the gaps in female-specific anatomy, but there is still much work to be done (e.g., Ah-King et al. 2014; Orbach et al. 2018; Hayssen and Orr 2020).

## Conclusion

Comparative Vertebrate Anatomy is a vibrant and active field of biology that continues to evolve. It is a field that is relevant to everyone, not just those interested in biology and the health fields. As such, laying the groundwork for best pedagogical practices is vital. Included in those practices is acknowledging and interrogating the influence of heteronormative white supremacist structures on our field and considering our role in undoing these norms.

The ideas contained in this paper are not solely those of the three authors; rather, this was a large effort by numerous practitioners of Comparative Vertebrate Anatomy. Our job, as authors, was to distill those efforts into a comprehensive manuscript. Given all of the above, we see this paper as an idea-generating document and not as an exhaustive and definitive list. We invite readers to continue thinking and discussing these topics with the intention of revisiting and refining the core concepts and competencies periodically. It is our hope that when the “second edition” of these core concepts and competencies is published, the grand challenges will be well integrated into the concepts and competencies.

## Supplementary Material

obac019_Supplemental_Files

## Data Availability

The data underlying this article are available in the article and in its online supplementary material.
