# Supplementary material for: The Core Concepts, Competencies, and Grand Challenges of Comparative Vertebrate Anatomy and Morphology
Source: Integr Org Biol. 2022 Jul 30;4(1):obac019. doi: 10.1093/iob/obac019 (PMC9338813; doi:10.1093/iob/obac019)
Supplement: obac019_Supplemental_Files [file obac019_supplemental_files.zip › Danos_Staab_Whitenack_2022_Supplement1.docx]

Supplement 1: A teaching guide

Table 1. The core concepts of Vertebrate Comparative Anatomy.

| **Core concept:**   1. Evolution   **Foundational components:**  The diversity, variation and unity of vertebrate anatomy is explained by descent with modification. | **Intrinsic ideas:**   1. Natural selection acts on variations of the phenotype, which determines functional performance and ultimately influences survival. 2. Homologous structures are similar morphological features found in different species that are a result of phylogenetic ancestry. 3. Analogous/homoplasic structures are similar morphological features in unrelated species that are a result of convergent evolution. 4. An animal’s form is constrained by its evolutionary history. This phylogenetic inertia distinguishes/unites the morphology of a given species and/or lineage. 5. The diversity of vertebrate forms is due to descent with modification and is evident through vertebrate evolutionary relationships/phylogeny. Therefore, vertebrate phylogenies provide frameworks for understanding vertebrate anatomy.   **Elaboration:**   1. Vertebrate phylogenies allow testing of hypotheses about ancestral vs. derived traits. 2. Major transformations in vertebrate evolution show how a newly evolved structure opens a new functional niche, allowing for potential rapid speciation (e.g., fins to limbs, agnathans to gnathostomes, evolution of flight). 3. Major transformation in vertebrate anatomy can be traced in the fossil record. 4. Old structures are co-opted or repurposed for new functions (exaptation) 5. Natural history observations provide the framework for evaluating evolutionary hypotheses. |
| --- | --- |
| **Core concept:**  B. Structure and Function  **Foundational components:**  The structure of vertebrate anatomy is heavily determined/ under heavy selection to match its functional demands; but because demands change over time and evolution acts on what already exists, an anatomical component at any given time may not have an optimal structure for its current function. | **Intrinsic ideas:**   1. Structure-function relationships are found at every level of the anatomical hierarchy (cells, tissues, organs, organ systems, animals). 2. Vertebrate anatomical structure is bound by the laws of physics. 3. Structure-function relationships can be constrained by phylogenetic ancestry and/or developmental pathways. 4. Anatomical diversity evolved with functional trade-offs or compromises in alternate versions of form. 5. As complexity increases [with more parts functioning together], there can be many structural solutions to a single functional problem.   **Elaboration:**   1. Laws of force and motion (including those related to fluid dynamics) are relevant to animal movement and hence structure of vertebrate anatomy. 2. Scaling and size–area volume relationships can explain many structure-function relationships. 3. Biological materials: elasticity, strength, toughness, etc. 4. Principles of simple machines (e.g. levers, beams, pulleys, etc.) are particularly useful for the study of the musculoskeletal system. 5. Bioinspired design: anatomical adaptations of vertebrate animals inform engineers, architects, and inventors in designing devices. |
| **Core concept:**  C. Morphological Development  **Foundational components:**  Vertebrate anatomy is expressed as phenotypes that are the result of genotypes executed through a developmental program. Major shifts in phenotype can be achieved through modularity which allows certain aspects of the phenotype to undergo major variations yet remain integrated in other ways. | **Intrinsic ideas:**   1. An organism’s phenotype is the expression of its genotype, which includes toolkit genes that program developmental pathways during embryogenesis and organogenesis. 2. Embryology, morphogenesis, organogenesis: much of the morphological variation among closely related groups can be understood as the outcome of common developmental processes. 3. In general, developmental pathways become increasingly complex as embryogenesis progresses and reflect the nested shared 4. The way that vertebrate embryos develop provides a conceptual basis for understanding most vertebrate structures. 5. Segmentation, an organizing principle of vertebrate bodies, is foundational to the diversification of serially homologous structures. 6. Organogenesis of complex morphologies can occur via developmental modules that become structurally or functionally integrated in an adult and may covary over evolutionary time. Alternatively, an integrated part can become released from its constraint and this allows that module to be decoupled and change independently from the rest. This is a foundational principle for the field of evolutionary developmental biology (evo-devo).   **Elaboration:**   1. There is a genomic basis for adaptation, as most phenotypes are determined by more than one gene. 2. Epigenetics is the underlying cellular and molecular mechanism for phenotypic plasticity. 3. The same genotype can produce different phenotypes, based on the environmental pressures during development (phenotypic plasticity). 4. Somites, myomeres, pharyngeal arches are examples of segmented developmental modules that are universal to vertebrate body plans. 5. Diversity in vertebrate cranial morphology is in part due to its developmental patterning via integrated modules. |
| **Core concept:**  D. Integration  **Foundational components:**  Anatomical structures develop, function and evolve as modules. These processes occur across space, time and biological levels of organization. | **Intrinsic ideas:**   1. Anatomical structures do not develop or function in isolation but as part of a whole organism. 2. Organisms do not live in isolation but as part of populations, species, communities and ecosystems. 3. Organisms are parts of nested networks of lineages. 4. As a result of A-C, anatomical structures develop, function and evolve as modules. 5. Modules can become structurally and/or functionally decoupled, or released from the constraints of their integrated units, and can subsequently evolve on their own.   **Elaboration:**   1. Ecology determines functional demands, and hence applies selection on anatomical structures. 2. The function of an anatomical structure may be redundant (many-to-one mapping) or constrained by multiple functional requirements. 3. Evidence from genetics, developmental biology, functional morphology, ecology and evolution must be integrated and interpreted together to understand the evolutionary history of vertebrate anatomy. |
| **Core concept:**  E. Human anatomy is the result of vertebrate evolution  **Foundational components:**  As vertebrate animals, human form has been constrained by phylogenetic ancestry. | **Intrinsic ideas:**   1. Vertebrate evolution is not linear and mammals are not at the top of vertebrate phylogenies. 2. The organization, function, and adaptive strengths and weaknesses of the human body have been shaped by evolutionary history. 3. The structural design and functional performance of some human anatomical parts is less than optimal due to phylogenetic constraint and/or functional compromises.   **Elaboration:**   1. A classic example of suboptimal design is the left recurrent laryngeal nerve. In humans, the left recurrent laryngeal nerve takes a longer, less efficient route than it does on the right side, due to the evolutionary history of the aortic arches. 2. In the eye: light travels through the nervous tissue to reach the photoreceptors in the back of the retina. |

Table 2. The core competencies/skills of Vertebrate Comparative Anatomy.

| **Core competency:**  F. Ability to apply tree thinking to the study of comparative vertebrate anatomy  **Foundational components:**  Interpretation and application of phylogenetic trees is crucial to the study of vertebrate anatomy | **Intrinsic ideas:**   1. Anatomical structures are not created de novo, but rather inherited from an ancestor. Thus, their interpretation requires an appreciation of ancestor-descendant relationships of vertebrates, as well as their phylogenetic relationships more broadly. 2. Vertebrate evolution is not linear, but rather branching 3. Phylogenies are hypotheses to which new data is added regularly 4. Phylogenies allow for reconstruction of evolutionary history, showing ancestral vs. derived species and traits   **Elaboration:**   1. Understanding phylogenetics provides lenses for understanding the diversity of structures we see in vertebrates 2. Understanding phylogenetic relationships among vertebrates provides a framework to explain the use of representative species in comparative anatomy |
| --- | --- |
| **Core competency:**  G. Ability to apply the skills of observation to the study of anatomical form  **Foundational components:**  The ability to make and record detailed, informative observations is an important skill for any scientist. | **Intrinsic ideas:**   1. Observation of specimens is an active venture and gives the raw materials for synthesis and creative interpretation.. 2. Small details can give us information about function and identification, distinguish species, identify what bone you’re looking at, describe a new species, or even notice something new that leads to a brand-new area of study. 3. Recording that rich detail is necessary for effective communication among individuals, disciplines and over time.   **Elaboration:**   1. The shape and positioning of a particular process on a bone may help determine which bone it is, which side of the body the bone came from, or which species the bone belongs to. 2. Small details, such as the number of gill rakers, can be used to distinguish species of fish that look superficially identical. |
| **Core competency:**  H. Ability to effectively dissect specimens  **Foundational components:**  Revealing structures in anatomical specimens requires careful dissection to preserve, not destroy, the structures of study. | **Intrinsic ideas:**   1. Tissues respond differently to forces and students will develop tactile knowledge of various tissues with practice. 2. Dissection tools are used for different outcomes. Blunt tools maintain integrity of structures. Sharp tools are used minimally with forethought on preserving adjacent structures. 3. Learning the three-dimensionality and integration of the vertebrate body is itself a three-dimensional, tactile process.   **Elaboration:**   1. During muscle dissection, superficial muscles are bisected and reflected approximately at the midpoint between origin and insertion. 2. Dissecting out blood vessels requires a more delicate touch than examining digestive organs, for example. 3. Two and three-dimensional photographic atlases of anatomy are not adequate for learning 3D anatomy. |
| **Core competency:**  I. Ability to depict anatomy  **Foundational components:**  Imaging (2D and 3D), creation of schematics, and descriptions of structures render observations of specimens for broad access and future study. | **Intrinsic ideas:**   1. Photographs in 2D do not always capture depth. 2. Medical imaging in 3D is also used for study of comparative vertebrate anatomy 3. Simplified schematics may convey observations more clearly than an image. 4. Drawing-to-learn has been shown to improve factual, inferential, and transfer learning (Cromley et al. 2020)   **Elaboration:**   1. CT scanning can lead to insights about the anatomy and function of delicate and small structures. 2. The names of blood vessels between branching points are constant, but the path between those branching points can be plastic between individuals (e.g., arteries in the digestive system originate from the aorta and venous drainage collects in the hepatic portal vein but branching to/from individual organs may vary slightly). A simple pathway diagram can eliminate confusion. |
| **Core competency:**  J. An appreciation of the importance of natural history collections  **Foundational components:**  The study of morphological evolution requires data that span space and time. Collections curate such data and make them available to students and scientists, speeding up the pace of scientific discovery. | **Intrinsic ideas:**   1. The diversity of morphological forms, in space and time, is held in natural history collections, primarily housed in and disseminated from museums but also may be available at home universities. 2. There is value in natural history collections that students need to appreciate to fully understand how progress is made in comparative anatomy.   **Elaboration:**   1. The increased digitization of museum specimens makes them a rich teaching resource. 2. Recent collections of 3-dimensional scans (e.g. forelimb comparisons on Sketchfab) provide a comparative anatomy resource for teachers and students. 3. The 3D digital models that are freely shared through resources like Morphosource.org and Sketchfab.com make it possible to 3D print rare specimens at relatively low cost. |
| **Core competency:**  K. The ability to communicate scientific information to peers and the public  **Foundational components:**  Teaching is one of the most effective forms of learning. By practicing how to communicate complex hypotheses about the origin and evolution of vertebrate morphology, students not only learn the material better but will be better equipped for sharing their knowledge. | **Intrinsic ideas:**   1. There are many misconceptions regarding how evolution works, many of them obvious in the familiar vertebrate anatomical systems.   **Elaboration:**   1. General public misunderstanding of the importance of funding basic vertebrate morphological research can be avoided with student understanding and communication of its core concepts broadly and effectively. |
| **Core competency:**  L. The ability to integrate data from multiple subdisciplines, applied to vertebrate anatomy  **Foundational components:**  The ability to ask for, evaluate, and integrate evidence from different subdisciplines improves critical thinking and the understanding of science as a multidimensional integrative endeavor. | **Intrinsic ideas:**   1. Morphological evolution is an inherently integrative process that acts across space, time and anatomical levels of organization. 2. Multiple types of evidence are required to rebuild the history of morphological evolution.   **Elaboration:**   1. Understanding how to read and interpret evidence from embryology, paleontology, and comparative biology is necessary for evaluating hypotheses regarding morphological evolution. 2. Can be aided by reading primary literature, which in itself is a necessary skill for biology students. |
