# Supplementary material for: The Core Concepts, Competencies, and Grand Challenges of Comparative Vertebrate Anatomy and Morphology
Source: Integr Org Biol. 2022 Jul 30;4(1):obac019. doi: 10.1093/iob/obac019 (PMC9338813; doi:10.1093/iob/obac019)
Supplement: obac019_Supplemental_Files [file obac019_supplemental_files.zip › Danos_Staab_Whitenack_2022_Supplement2.docx]

Supplement 2: Teaching Resources

Table 1. Online resources

| **Core Concept/ Competency** | **Resource Name** | **URL** | **Description** |
| --- | --- | --- | --- |
| A,C,D,E,K,L | PBS learning - Your Inner Fish | <https://www.biointeractive.org/classroom-resources/your-inner-fish> | Streaming video of Your Inner Fish series |
| A,F | NOVA Labs Evolution Lab | <https://www.pbs.org/wgbh/nova/labs/lab/evolution/> | Teaches tree thinking in an online game format. Requires free account to be made. |
| A,F | Understanding Evolution (Berkley) | <https://evolution.berkeley.edu/evolibrary/home.php> | "The Tree Room" lesson on tree thinking, undergraduate teachers' lounge, misconceptions about evolution |
| B | Animal Locomotion lecture videos | <https://mchenrylab.bio.uci.edu/e139/> | Course on "Animal Locomotion" by Manny Azizi and Matt McHenry at UC Irvine. This serves as an introductory biomechanics course. Problem sets available upon request (mmchenry@uci.edu). |
| B | Awesome Biomechanics | <https://github.com/modenaxe/awesome-biomechanics> | Has a variety of sources - teaching resources, datasets, online lectures, and more |
| B | Virtual Microscopy - Yale | <https://virtualmicroscopy.peabody.yale.edu/results.php?t=Comparative+Anatomy> | Collection of histological slides |
| B | Zoological Motion Analysis Research Portal | <http://zmaportal.org/zmaportal/larequest.php?request=alldata&fbclid=IwAR2s1as4h9xnDX_EHgHner8o9kIwIR1neHAxBVi69IQTfAX6uDwQT5EHqIk> | Videos of animals in motion |
| B,E,G | eSkeletons | <http://www.eskeletons.org/> | Online source for primate skeleton comparison |
| B,I | 3D models from Lauridsen et al. 2016 | <https://doi.org/10.5061/dryad.8787m> | Series of 3D models, including ready-to-print, from Lauridsen et al.'s 2016 paper |
| B,I | Digimorph | <http://digimorph.org/index.phtml> | CT scans of vertebrates, mostly skeletal |
| B,I | Morphosource | <https://www.morphosource.org/> | 3D models of biological material, mostly skeletal |
| B,I | Oregon State scanned specimens | <https://sketchfab.com/osuecampus/collections/fw-scan-project> | Variety of scanned vertebrate specimens |
| B,I | oVERT | <https://www.floridamuseum.ufl.edu/science/overt/> | Collection of 3D vertebrate anatomy models |
| B,I | Sketchfab - BI 315 Comparative Vertebrate Anatomy | <https://sketchfab.com/billryerson/collections/bi-315-comparative-vertebrate-anatomy> | Collection of models specifically for vertebrate anatomy |
| B,I | Sketchfab - Blackurn Lab | <https://sketchfab.com/ufherps/collections/vertebrate-anatomy-skeletons> | Collection of models specifically for vertebrate anatomy |
| B,I | Witmer Lab - 3D visualizations | <https://people.ohio.edu/witmerl/3D-Visualization.htm> | QuickTime movies & 3D PDFs of CT scanned vertebrates |
| B,I,J | Smithsonian Open Access | <https://www.si.edu/openaccess> | 2D and 3D models from the Smithsonian museums. 3D models are mostly mammal bones, but there are also some dinosaurs. |
| G | Science Observation Activity | <https://serc.carleton.edu/sp/process_of_science/examples/sci_observe.html> | Simple activity where students observe a process in their lives and practice using observation to answer questions about the process |
| H | All About Bird Anatomy from Cornell University | <https://academy.allaboutbirds.org/features/birdanatomy/> | Interactive illustrations of generalized bird anatomy |
| H | Kenyon College Cat Anatomy Tutorial | <https://biology.kenyon.edu/heithausp/cat-tutorial/welcome.htm> | Labeled, online photos of cat skeleton and internal organs |
| H | Murray State Comparative Anatomy Atlas | <http://campus.murraystate.edu/academic/faculty/tderting/anatomyatlas/> | Mix of labeled and unlabeled photos of various vertebrates |
| H | PBS Learning - Dissection 101 | <https://wqln.pbslearningmedia.org/collection/dissection-videos-for-classroom-use/?fbclid=IwAR2XUfCjbkHcPPQ6aaPLXfrbco2YmiNMMQDAa7W-g4DUQqOFYECYArCIYnA#.Xs6tky-z1bU> | Videos of dissections: dogfish, sheep heart, cow eye, frog, perch |
| H | Reynolds (1897): The Vertebrate Skeleton | <http://gutenberg.readingroo.ms/4/3/4/3/43431/43431-h/43431-h.htm> | Free e-book from The Project Gutenberg with really wonderful illustrations |
| H | Savalli's Snake Skulls | <http://www.savalli.us/BIO370/Anatomy/5.SnakeSkullLabel.html> | Labeled and unlabeled snake skull photos |
| H | Whiteman College - virtual pig dissection | <https://www.whitman.edu/academics/majors-and-minors/biology/virtual-pig> | Series of labeled photos that step through dissecting fetal pigs |
| General | CourseSource | <https://www.coursesource.org/> | Variety of peer-reviewed teaching resources |
| General | HHMI BioInteractive | <https://www.biointeractive.org/> | Variety of videos and activities |
| General | Life Science Teaching Resource Community | <https://www.lifescitrc.org/index.cfm> | Variety of teaching resources |
| General | National Center for Case Study Teaching in Science | [https://sciencecases.lib.buffalo.edu/](https://sciencecases.lib.buffalo.edu/?fbclid=IwAR2RS9SHHSwoHcnGdWJ6Fn2SnKA_BRIHwnImtxinU0fMFCGuoG-DbtPcZDQ) | Wide range of case studies across multiple disciplines. Includes undergraduate levels |
| General | National Center for Case Study Teaching in Science | <https://sciencecases.lib.buffalo.edu/> | Collection of case studies, includes both medical-oriented and evolution-oriented anatomy cases |

Table 2. Publications

| **Core Concept/ Competency** | **Resource** | **Citation info** | **URL or ISBN** | **Description/More Information** |
| --- | --- | --- | --- | --- |
| A,B,C,D | Dial et al. 2015 | Dial KP, Shubin N, Brainerd EL. 2015. Great Transformations in Vertebrate Evolution. Chicago: University of Chicago Press. | 978-0226268255 | Each chapter deals with a major vertebrate evolutionary innovation or transformation (e.g. feathers, limbs, neck) |
| A,C,D,E,K,L | Shubin 2008 | Shubin N. 2008. Your Inner Fish: A Journey into the 3.5-billion-year History of the Human Body. New York: Vintage Books. | 978-0307277459 | Popular science book that focuses on the water-land vertebrate transition and human anatomy |
| A,F | Baum 2005 | Baum DA, DeWitt Smith S, Donovan SSS. 2005. The tree-thinking challenge. Science. 310:979-980. | <https://science.sciencemag.org/content/310/5750/979.full> | A short perspectives paper on the importance of tree-thinking, which also includes 2 quizzes |
| A,F | Eddy et al. 2013 | Eddy SL, Crowe AJ, Wenderoth MP, Freeman S. 2013. How should we teach tree-thinking? An experimental test of two hypotheses. Evol Educ Outreach. 6:1-11. | <https://evolution-outreach.biomedcentral.com/articles/10.1186/1936-6434-6-13> | Describes evidence for having students build trees in order to learn tree-thinking, and suggests learning outcomes. |
| A,F | Whitenack and Drew 2018 | Whitenack LB, Drew JA. 2019. Untangling the contribution of characters to evolutionary relationships: a case study using fossils, morphology, and genes. J Biol Educ. 53:271-224. | <https://doi.org/10.1080/00219266.2018.1469533> | Outlines a class activity that has students build trees in PAST and compare trees built from molecular, morphological, and fossil data. |
| B,I | Lauridsen et al. 2016 | Lauridson H, Hansen K, Nørgård MØ, Wang T, Pedersen M. 2016. From tissue to silicon to plastic: three-dimensional printing in comparative anatomy and physiology. R Soc Open Sci. 3:150643. | <https://royalsocietypublishing.org/doi/10.1098/rsos.150643> | Short publication on how to use 3D printed models and the workflow from CT & MRI to models for vertebrate anatomy and physiology |
| G | Ainswoth et al. 2011 | Ainsworth S, Prain V, Tytler R. 2011. Drawing to learn in science. Science 333:1096-1097. | <https://doi.org/10.1126/science.1204153> | Short paper on why drawing is important for the scientific process and communication. |
| G | Look at your fish! | Scudder, SH. 1874. In the laboratory with Agassiz. Every Saturday. 6:369-370. | <https://www.thoughtco.com/look-at-your-fish-by-scudder-1690049> | A portion of Scudder's essay stresses the importance of observation and drawing in natural history. Author LBW uses this as a stepping point for a lab focused on these competencies |
| G | Moore et al. 2011 | Moore CM, Lowe C, Lawrence J, Borchers P. 2011. Developing observational skills and knowledge of anatomical relations in art and anatomy workshop using plastinated specimens. Anat Sci Educ 4:294-301. | <https://doi.org/10.1002/ase.244> | Describes workshops that paired art and medical students together to draw and observe anatomical structure |
| G | Oguz and Yurumezoglu 2007 | Oguz A, Yurumezoglu K. 2007. The primacy of observation in inquiry-based science teaching. International Workshop Science Education in School. Oct. 11-14, 2007. | <https://files.eric.ed.gov/fulltext/ED498653.pdf> | Describes the results of a study centered around students using systematic observation |
| Grand Challenge | Cronin et al. 2021 | Cronin MR, Alonzo SH, Adamczak SK, et al. 2021. Anti-racist interventions to transform ecology, evolution and conservation biology departments. Nature Ecol Evol. 5:1213-1223. | <https://doi.org/10.1038/s41559-021-01522-z> | Describes a toolkit of interventions to foster anti-racism in the classroom, in research laboratories, and department-wide |
| Grand Challenge | Schinske et al. 2017 | Schinske JN, Perkins H, Snyder A, Wyer M. 2017. Scientist Spotlight homework assignments shift students' stereotypes of scientists and enhance science identity in a diverse introductory science class. CBE LIfe Sci Educ. 15:ar47. | <https://doi.org/10.1187/cbe.16-01-0002> | Describes "Scientist Spotlight" assignments, which feature "counterstereotypical examples of scientists" |
| Grand Challenge | Cooper et al. 2020 | Cooper KM, Auerbach AJJ, Bader JD, et al. 2020. Fourteen recommendations to create a more inclusive environment for LGBTQ+ individuals in academic biology. CBE Life Sci Educ 19:es6. | <https://doi.org/10.1187/cbe.20-04-0062> | Contains recommendations for teaching biology across disciplines and levels in a way that is inclusive for LGBTQ+ folks |
| Grand Challenge | *Anatomical Record* 35(4), April 2022 | N/A | <https://anatomypubs.onlinelibrary.wiley.com/toc/19328494/2022/305/4> | Special issue that is focused on the history and future of human anatomy with regard to diversity, equity, inclusion, and justice |
| Grand Challenge | Štrkalj and Pather 2021 | Štrkalj G, Pather N. 2021. Beyond the sex binary: Toward the inclusive anatomical sciences education. *Anat Sci Educ* 14:513-518. | <https://doi.org/10.1002/ase.2002> | Contains suggestions for teaching reproductive anatomy in a non-binary way |
