# Supplementary material for: The Core Concepts, Competencies, and Grand Challenges of Comparative Vertebrate Anatomy and Morphology
Source: Integr Org Biol. 2022 Jul 30;4(1):obac019. doi: 10.1093/iob/obac019 (PMC9338813; doi:10.1093/iob/obac019)
Supplement: obac019_Supplemental_Files [file obac019_supplemental_files.zip › Danos_Staab_Whitenack_2022_FigureTableCaptions.docx]

Figure 1. Graphical representation of the core concepts of comparative vertebrate anatomy, highlighting the integration of all core concepts. Any given part of vertebrate anatomy is inherently integrative (Core Concept D) because it is determined by three axes: interactions in space (spatial interactions), time and level of biological organization (anatomical level). Spatial interactions span cell-to-cell to ecosystem level interactions. Although further spatial interactions are possible, they are likely rare and unlikely to have had an effect on anatomical evolution. Temporal factors range from a few hours, the time it takes for the anatomy to develop embryologically, to millions of years, the amount of time represented in the fossil record. Vertebrate anatomy is the sum of evolutionary modifications at multiple levels of biological organization, termed here anatomical level. Therefore, Comparative Vertebrate Anatomy can be studied anywhere along the continuum of these three axes. The triangle formed by the apices of the three axes represents Evolution (Core Concept A). Structure-function (Core Concept B), acts across all anatomical levels to give rise to vertebrate anatomy but within a narrower range of spatial interactions and time. Structure-function for anatomical structures is not possible before a structure is formed hence this concept does not encompass individual cell-to-cell interactions. Similarly, at higher complexity spatial interactions each interaction is likely to have a negligible effect on the structure-function relationship of a single anatomical structure. Structure-function is also unlikely to be a significant factor during development because the structure is not yet fully formed. Development (Core Concept C) is true for all anatomical levels, from genes to individuals, with influence from spatial interactions that fade near the inter-individual level and at the time span of an animal’s lifespan. Structure-function and development overlap because structure cannot evolve without developmental pathways changing.

Supplement 1- Teaching Guide

Table 1. The core concepts of Vertebrate Comparative Anatomy.

Table 2. The core competencies/skills of Vertebrate Comparative Anatomy.

Supplement 2- Resources for teaching core concepts and competencies

Table 1. Online resources

Table 2. Publications
